# Excision of Solitary Non-syndromic Oral Plexiform Neurofibroma Utilizing a Diode Laser: A Case Report

**DOI:** 10.7759/cureus.55277

**Published:** 2024-02-29

**Authors:** Mohammed M Al-Ali, Lubna M Al-Otaibi, Ibtissam Al-Bakr

**Affiliations:** 1 Oral Medicine and Special Care Dentistry, Prince Sultan Military Medical City, Riyadh, SAU; 2 Oral and Maxillofacial Surgery, King Fahad Hospital, Hofuf, SAU

**Keywords:** neurofibromatosis, diode laser, solitary, alveolar ridge, plexiform neurofibroma

## Abstract

Plexiform neurofibroma is a benign peripheral nerve sheath tumor known to be pathognomonic for neurofibromatosis type 1. However, solitary plexiform neurofibroma in the oral cavity is extremely rare. Herein, we presented a 73-year-old Saudi male with solitary plexiform neurofibroma located on the maxillary alveolar ridge, which was excised successfully using a 940 nm diode laser. Microscopic examination revealed a multinodular arrangement of benign spindle cells in a haphazard pattern. Immunohistochemical analysis showed positive staining for S100 and CD34 in the tumor cells.

## Introduction

The World Health Organization classification defines neurofibroma (NF) as "a benign peripheral nerve sheath tumor consisting of differentiated Schwann cells, perineurial/perineurial like cells, fibroblasts, mast cells, and residual interspersed myelinated and unmyelinated axons embedded in a myxoid and collagenous extracellular matrix" [1]. It is considered the most common peripheral nerve sheath tumor that can appear as a solitary lesion or multiple lesions as a part of neurofibromatosis syndrome, most commonly neurofibromatosis type 1 (NF-1). NF commonly involves the cutaneous area, with the majority occurring as solitary lesions [1].

Solitary NF is known for its benign behavior and slow progression, somewhat circumscribed but a non-encapsulated tumor. Diagnosis of solitary NF relies on the absence of other features related to the systemic disease of neurofibromatosis. Intraorally, the presence of solitary NF is exceedingly uncommon [2].

According to prior literature, the prevalence of solitary NF in the oral cavity is estimated to be around 6.5%, particularly in cases unrelated to NF-1. The tongue and buccal mucosa are frequently affected intraorally, although these tumors can arise at any location, markedly the palate and floor of the mouth. Furthermore, in the oral cavity, the occurrence of plexiform neurofibroma (PNF) is uncommon. PNF affects approximately 5-15% of individuals with NF-1. However, literature is scarce in documenting cases of solitary PNF that exclusively involve peripheral nerves without any accompanying characteristic manifestations of NF-1 [3-5].

Surgical excision is the treatment of choice for a solitary lesion, and it can be accomplished through conventional surgery, electrosurgery, or laser techniques. Throughout the procedure, utmost attention is given to preserving the nerve from which the tumor arises [6].

In this report, we describe a case of solitary non-syndromic PNF in an elderly male from Saudi Arabia. The tumor was located on the upper anterior alveolar ridge and was successfully excised using a diode laser, resulting in an excellent clinical outcome.

## Case presentation

**Figure 1:**
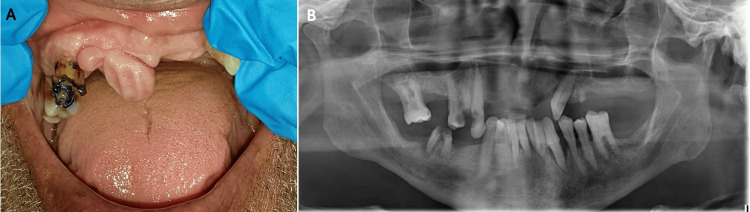
Intra-oral and radiographic examination (A) Intra-oral examination shows a well-defined solitary, bi-lobulated, firm, pedunculated exophytic lesion on the upper anterior alveolar ridge. (B) Panoramic radiograph showed no bone involvement

A 73-year-old diabetic and hypertensive male patient was referred with a chief complaint of a painless swelling in the upper anterior region of the mouth. He reported it to be present for many years. The intra-oral examination revealed a painless, solitary, bi-lobulated, oval, firm, pink, pedunculated, and exophytic soft tissue mass on the upper right anterior alveolar ridge, in the area of the incisors, extending to the mesial surface of the canine and measuring 1.2×1.7 cm. The panoramic radiograph showed no bone involvement (Figure 1A, 1B).

The extra-oral examination was unremarkable. A routine hematological examination revealed normal red and white blood cell and platelet counts. Coagulation and general profiles were within normal limits. The differential diagnosis included fibroma, peripheral ossifying fibroma, long-standing pyogenic granuloma, and schwannoma.

**Figure 2:**
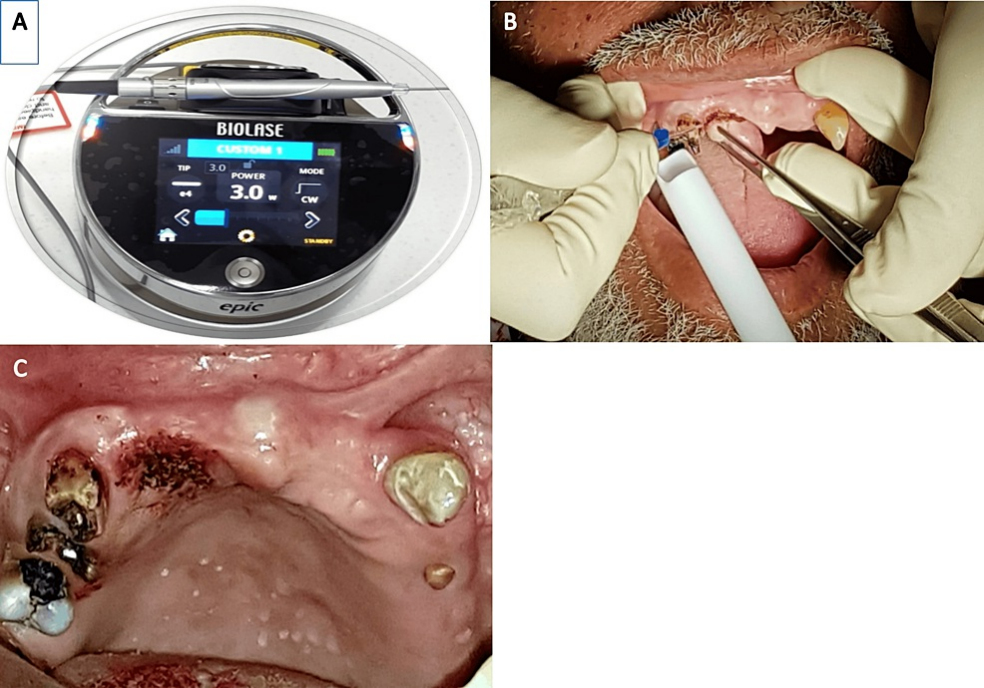
Photos during operation (A) Surgical excision setting. (B) Lesion during excision with diode laser. (C) Immediate postoperative view

Following the administration of local anesthesia (2% lidocaine with 1/100,000 epinephrine), the lesion was excised using a 940 nm diode laser (Epic Biolase, Irvine, California, United States) with an average power of 3.5 W, in a continuous wave (CW) mode. The initiated surgical laser tip (ezTip E3-4mm) was applied in contact mode with a focused beam and moved around the base of the lesion using the circumferential incision technique (CIT) to remove the tissue. Bleeding was stopped using the laser hemostasis setting (0.5 W, CW), and no suturing was needed (Figure 2A-2C).

**Figure 3:**
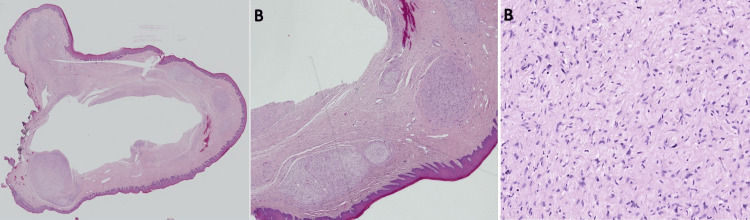
Hematoxylin and eosin-stained microscopic photographs of the lesion (A-B) Low magnification shows a submucosal lesion arranged in a multinodular and plexiform pattern (×2 and ×10). (C) Higher magnification shows Schwan and fibroblast cells arranged haphazardly in addition to mast cells (×20)

All required safety measures including wearing protective goggles and using high vacuum suction were applied throughout the procedure. The excised specimen was placed in 10% neutral buffered formalin and sent for histopathological examination. Microscopic examination revealed a submucosal lesion arranged in a multinodular and plexiform pattern. The nodules were composed of disorganized and haphazardly arranged spindle cells, one with elongated wavy and buckled nuclei (Schwann cells) and others with ovoid nuclei (fibroblast) with splayed apart collagen fibers with variable thickness throughout each nodule in myxo-collagenous stroma within a fibrovascular connective tissue (Figure 3A-3C).

**Figure 4:**
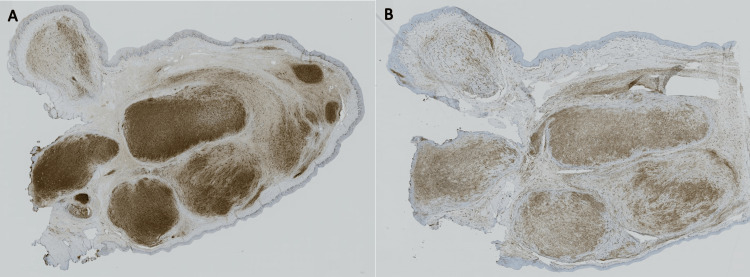
Immunohistochemical-stained microscopic photographs of the lesion (A) S100-stained Schwan cells. (B) CD34-stained fibroblast cells

The histopathologic differential diagnoses were leiomyoma, schwannoma, traumatic neuroma, and NF. Upon performing immunohistochemistry, the Schwann cells were positive for S100 and fibroblast cells for CD34, and both were negative for smooth muscle antibody (SMA) (Figure 4A, 4B).

Therefore, microscopic and immunohistochemical examinations indicated the diagnosis of PNF. The patient then underwent further investigations for other manifestations of NF-1, and no typical features of this syndrome were identified. After considering the previous information, the final diagnosis was established as a solitary PNF.

**Figure 5:**
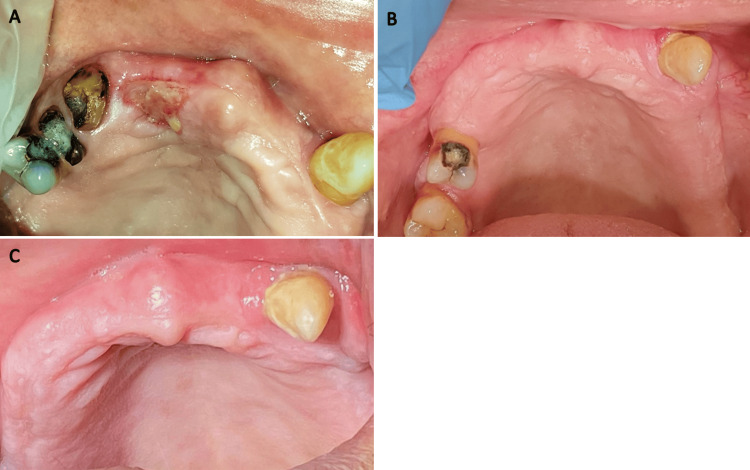
Postoperative intra-oral photographs of the operation site (A) One week. (B) Six months. (C) Thirty-six months

The laser-assisted surgical excision was found to be easy to perform with excellent precision and minimum bleeding. Moreover, it was well tolerated by the patient who also reported mild postoperative pain. Healing occurred within three weeks without any complications. The patient was then followed up for 36 months, and no recurrence was noted (Figure 5A-5C).

## Discussion

NF is the most common peripheral nerve sheath tumor that exhibits characteristic features, including the presence of a neural component consisting of transformed Schwann cells and nonneoplastic fibrous components that contain fibroblasts [1]. A PNF is identified by its unique growth pattern, presenting as a diffuse, elongated, or plexiform mass that affects multiple nerve fascicles [7].

The presence of PNF is highly indicative and pathognomonic for NF-1. NF-1 is an autosomal dominant inherited disease caused by a mutation in the NF1 gene located on chromosome 17. NF-1 exhibits a range of clinical features, varying significantly among affected individuals, including those within the same family. The main clinical features of NF-1 encompass café au lait macules, axillary and inguinal freckling, neurofibromas, optic glioma, Lisch nodules, and characteristic bone lesions like sphenoid dysplasia [7,8]. As PNF associated with NF-1 has the potential for malignant transformation [3], it is important to further investigate and follow-up these patients.

The occurrence of a solitary PNF in the oral cavity is a scarce condition, with only a few reported cases documented in the English literature [3-5,7-15]. Table 1 provides a summary of these cases.

**Table 1:** Clinicopathological and immunohistochemical features of solitary plexiform neurofibroma involving the oral cavity previously published in the literature including our case M: male; FL female; IHC: immunohistochemistry; NM: not mentioned

	Author	Age (in years)/sex	Country	Site	Clinical	IHC	Treatment	Follow-up
1	Our case	73/M	Saudi	Alveolar ridge	Painless, bi-lobulated, oval, firm, pink, pedunculated, and exophytic soft tissue mass, measured 1.2×1.7 cm	+ve S100 and CD34; -ve SMA	Excision using diode laser	36 months with no recurrence
2	Zhang et al. [9]	50/F	Chinese	Tongue	Painless, well-circumscribed rounded mass since many years ago in the left hemi-tongue		Conventional surgical excision	Six months with no recurrence
3	Rao et al. [3]	60/F	India	Tongue	Painless swelling for 3-4 years, pale pink in color, ovoid in shape, pedunculated, mobile and soft in consistency, measured 2×2 cm	+ve S100; -ve SMA	Conventional surgical excision	NM
4	Mahalle et al. [7]	37/F	India	Labial mucosa	Painless swelling for four years that gradually grew in size. The swelling was soft to firm in consistency	No	Conventional surgical excision	15 months with no recurrence
5	S et al. [5]	57/F	India	Upper gingiva	Well-circumscribed, painless, firm growth, bi-lobulated, pedunculated, measured 0.75-2 cm	No	Conventional surgical excision	12 months with no recurrence
6	Kınış et al. [4]	18/M	Turkey	Buccal mucosa	Firm, non-tender, mobile, non-pulsatile mass for 10-12 years, mild pain for six months, measured 3.5x1.5x2 cm	No	Conventional surgical excision	NM
7	Sharma et al. [10]	11/F	India	Tongue	Hypertrophy of the right side of the tongue, soft, non-tender, nonreducible, and non-pulsatile intact, measured 3×3×2.5 cm	No	Conventional surgical excision	10 months with no recurrence
8	Zwane et al. [11]	4/F	South Africa	Lower labial mucosa	Painless swelling of long duration, measuring 2 cm and resembling a double lip, non-pulsating, rubbery consistency	No	Conventional surgical excision	NM
9	Guclu et al. [12]	5/F	Turkey	Tongue	Inability to close the mouth because of a large, asymmetrical, protruding tongue	No	Conventional surgical excision	10 months with no recurrence
10	Marocchio et al. [8]	24/F	Brazil	Right buccal mucosa	Well-circumscribed, painless, firm, measured 1.1×1.8 cm	+ve S100, CD34, EMA (perineurium)	Conventional surgical excision	Three years with no recurrence
11	Lin et al. [13]	7/F	Canada	Tongue	Slowly growing mass in the right hemi-tongue	NM	Conventional surgical excision	At least for seven years with no recurrence
12	Gómez-Oliveira et al. [14]	67/F	Spain	Left buccal mucosa	Painless, slowly increasing in size, mobile, well-delimited, and elastically consistent, measured 2 or 3 cm	NM	Conventional surgical excision	Two years with no recurrence
13	Alatli et al. [15]	37/F	Turkey	Vestibular side of the right mandibular first premolar	Paresthesia on the oral side of her right lower lip after local anesthesia, firm, mobile mass, measured 1.5 cm in diameter	NM	Conventional surgical excision	NM

The majority of reported cases of oral PNF have been observed in the Asian population, with a higher prevalence among female patients. Remarkably, only two cases, including ours, have been reported in male patients. The age range of individuals affected by PNF spans from four to 73 years old; thus, our patient is the eldest among the reported PNF cases. The tongue was the most frequently affected oral site, followed by the buccal mucosa. Notably, our case was the only one that exhibited involvement of the upper alveolar ridge.

In most cases, conventional surgical excision has been the preferred treatment approach for PNF. However, in our particular case, we opted for the use of a diode laser. As PNF can be highly vascular, excessive bleeding may occur during conventional surgery, which thus can hinder the surgeon's visibility, prolong the operation time, and increase the risk of postoperative complications such as edema, pain, and healing time. The literature discusses several advantages of using a diode laser compared to conventional surgery, such as a decreased need for local anesthesia, hemostatic properties resulting in reduced intraoperative bleeding and suturing requirements, lesser operation and healing times, antimicrobial properties that help maintain a sterile environment, and minimization of postoperative pain and complications [16,17].

Except for a single case that presented with paresthesia of the lower lip, all other cases were reported to be asymptomatic. The underlying mechanism responsible for developing isolated, solitary PNF remains uncertain. Specific mentions have been made regarding trauma as a potential cause [7,18]. Both our case and case number 13 lend support to this hypothesis, as our case may have developed due to dental trauma, while the other case may have resulted from trauma caused by a dental needle during local anesthesia administration.

## Conclusions

This case report describes a solitary oral non-syndromic PNF. However, the patient managed here, unlike others, is an elderly Saudi Arabian male with a long-standing PNF appearing on the upper anterior alveolar ridge. The lesion was successfully excised using a diode laser with minimum bleeding and discomfort resulting in an excellent clinical outcome. Subsequently, the postoperative healing process proceeded without complications, and no recurrence of the lesion occurred.
